# Supplementary material for: Membrane Tension Integrates Physical and Signaling Cues to Gate Cell Fate Transitions
Source: bioRxiv. 2026 Mar 5:2026.03.04.708749. Preprint. [Version 1] doi: 10.64898/2026.03.04.708749 (PMC13001439; doi:10.64898/2026.03.04.708749)

bioRxiv preprint doi: https://doi.org/10.64898/2026.03.04.708749; this version posted March 5, 2026. The copyright holder for this preprint (which was not certified by peer review) is the author/funder, who has granted bioRxiv a license to display the preprint in perpetuity. It is made available under aCC-BY 4.0 International license.

Supplemental Figure 1, Ali and Gibbard et al

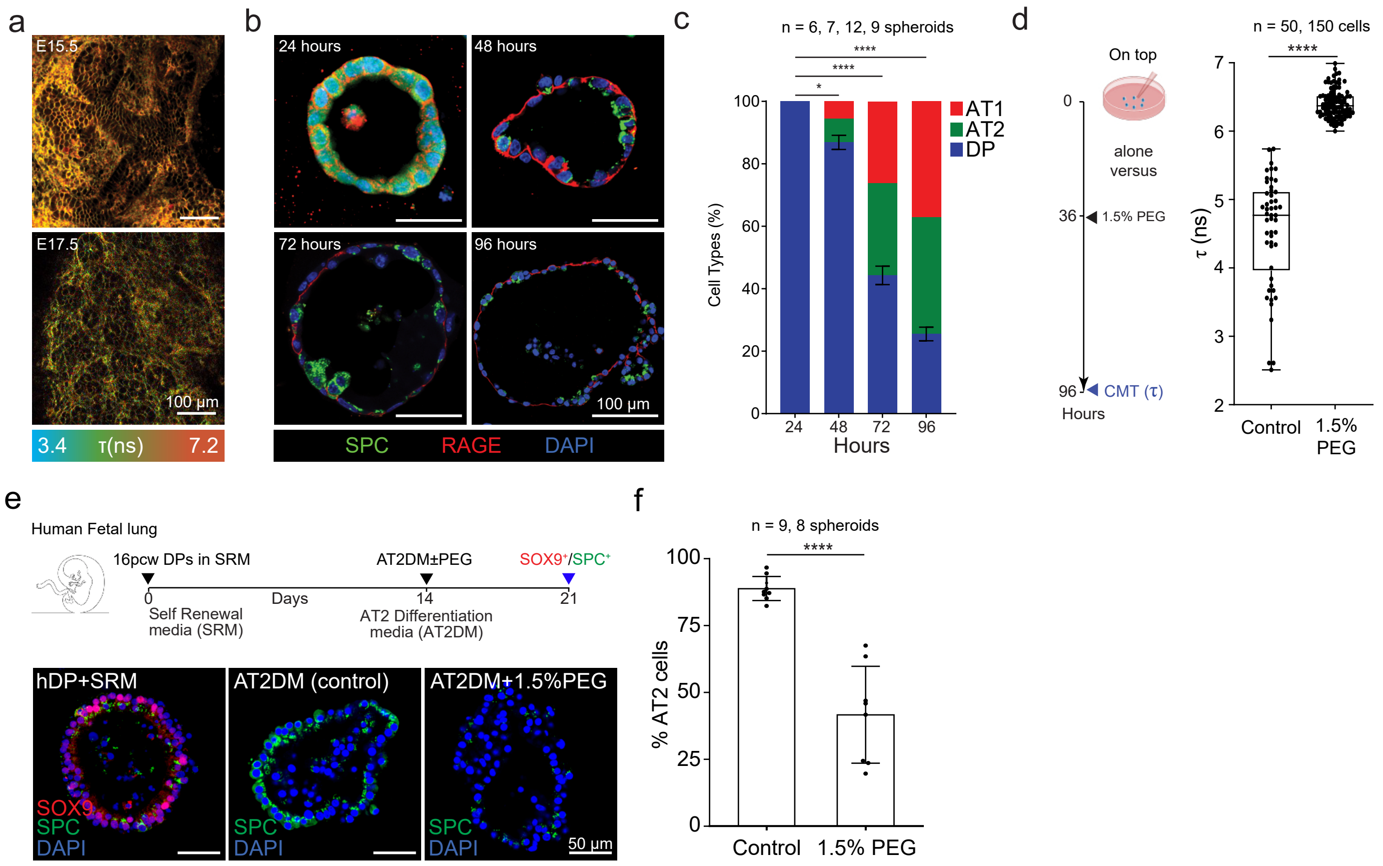

bioRxiv preprint doi: https://doi.org/10.64898/2026.03.04.708749; this version posted March 5, 2026. The copyright holder for this preprint (which was not certified by peer review) is the author/funder, who has granted bioRxiv a license to display the preprint in perpetuity. It is made available under aCC-BY 4.0 International license.

Supplemental Figure 2, Ali and Gibbard et al

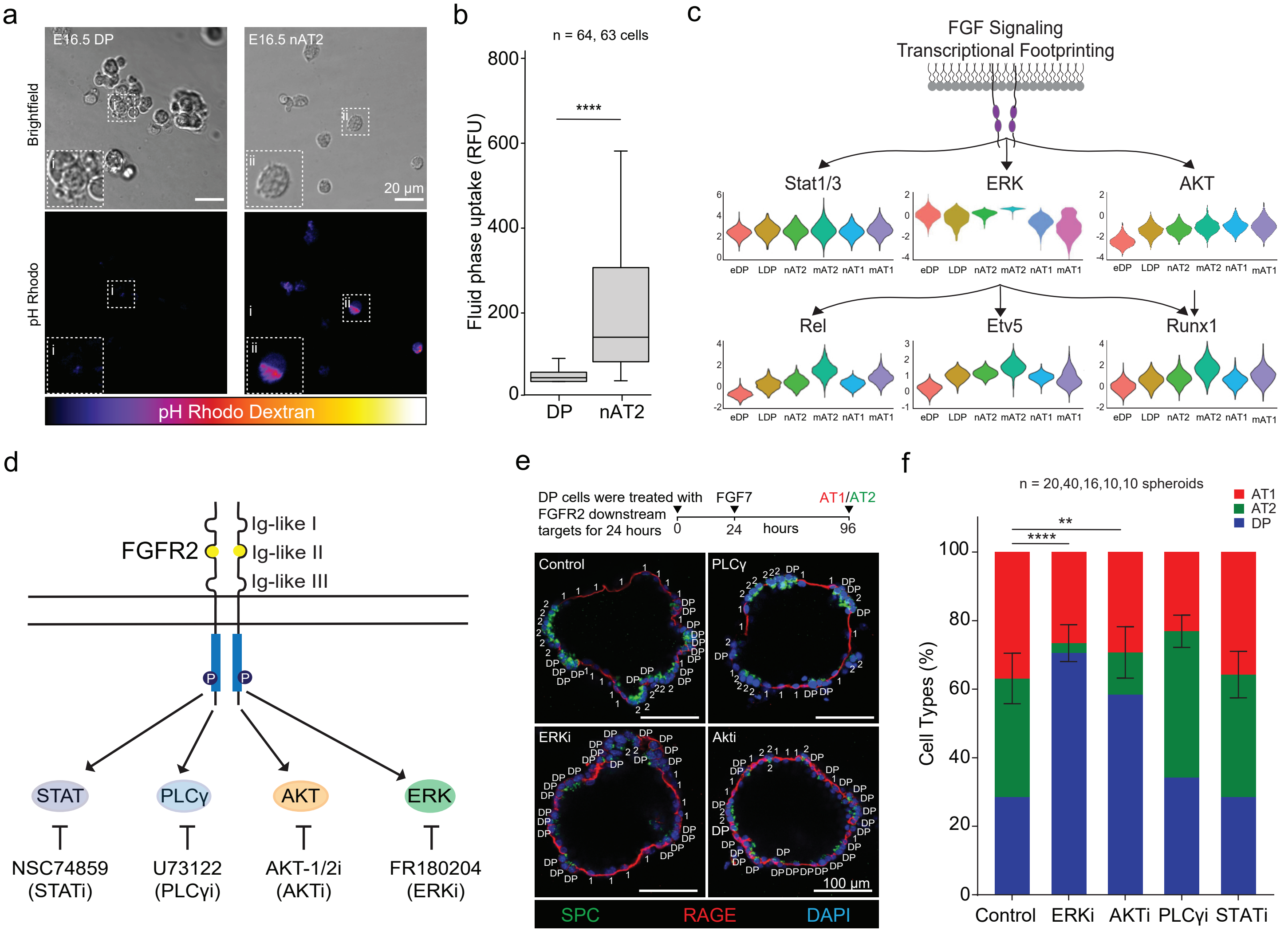

bioRxiv preprint doi: https://doi.org/10.64898/2026.03.04.708749; this version posted March 5, 2026. The copyright holder for this preprint (which was not certified by peer review) is the author/funder, who has granted bioRxiv a license to display the preprint in perpetuity. It is made available under aCC-BY 4.0 International license.

Supplemental Figure 3, Ali and Gibbard et al

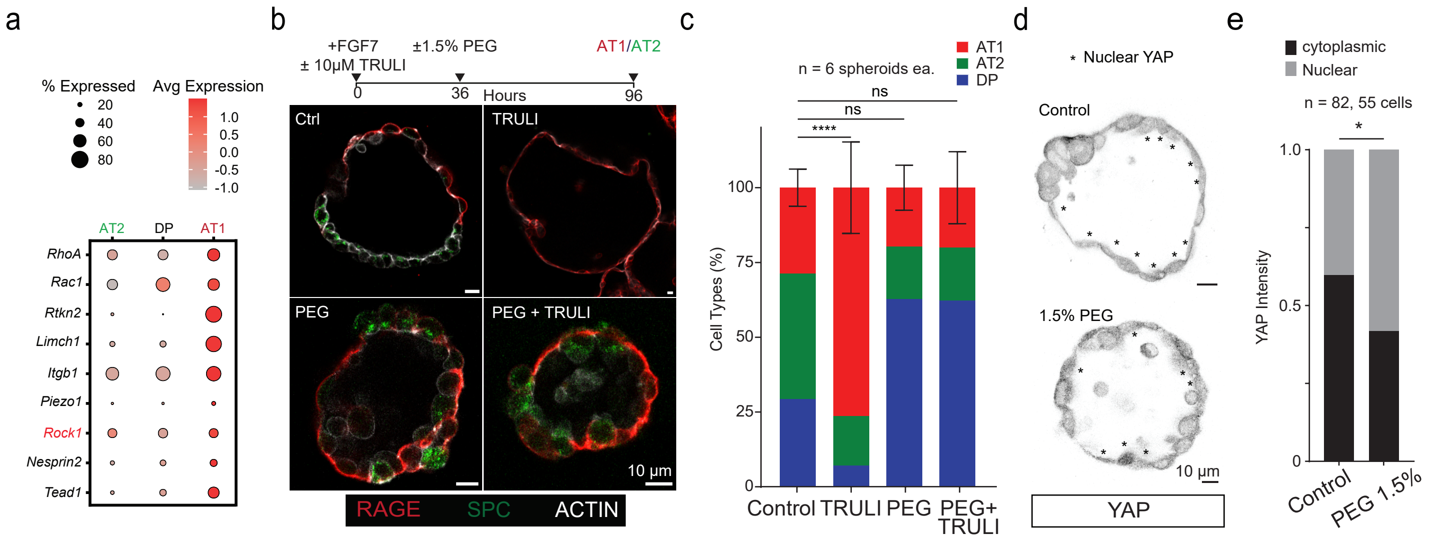

bioRxiv preprint doi: https://doi.org/10.64898/2026.03.04.708749; this version posted March 5, 2026. The copyright holder for this preprint (which was not certified by peer review) is the author/funder, who has granted bioRxiv a license to display the preprint in perpetuity. It is made available under aCC-BY 4.0 International license.

Supplemental Figure 4, Ali and Gibbard et al

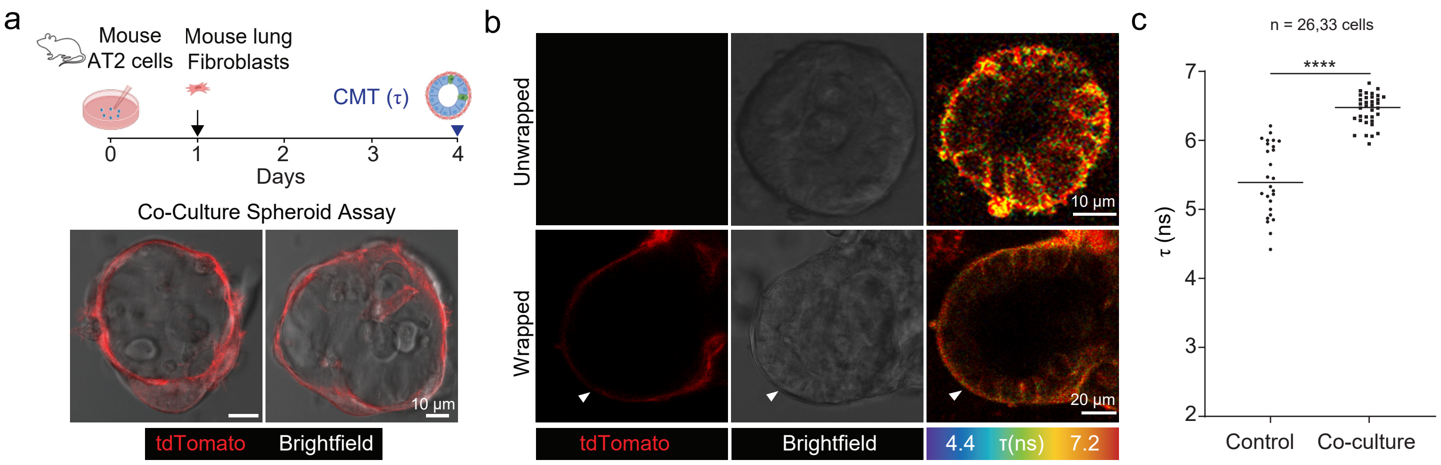

bioRxiv preprint doi: https://doi.org/10.64898/2026.03.04.708749; this version posted March 5, 2026. The copyright holder for this preprint (which was not certified by peer review) is the author/funder, who has granted bioRxiv a license to display the preprint in perpetuity. It is made available under aCC-BY 4.0 International license.

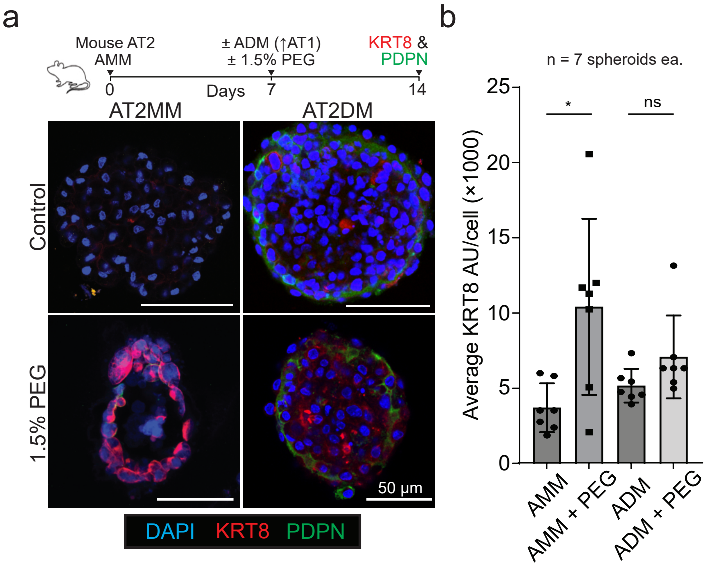

Supplement: 1 — Supplementary Figure 1: (A) Representative images of embryonic lung tissues at stages E15.5 and E17.5 stained with Flipper-TR. The scale bar represents 100μm. (B) Immunostained images show DP cultured on Matrigel for four days, with media supplemented every two days with 50ng/ml FGF7. Fixation occurred at 24-hour intervals: 24, 48, 72, and 96 hours. The fixed spheroids were stained for AT1 marker Rage (red), AT2 marker Sftpc (green), and nuclear DAPI (blue). Scale bars indicate 100μm. (C) Graph showing the percentage of DP cells per spheroid at 24-hour intervals (n = 10 spheroids for each condition, in triplicate). p values (*0.0145, **** <0.0001) were calculated using one-way ANOVA with multiple comparisons. (D) DP cell seeded on matrigel supplemented with 50ng/ml FGF7. Media was supplemented with 1.5% PEG300 to raise CMT. CMT was measured by τ-gating of PEG-treated spheroids vs control after 4 days. Data are shown as means ± SDs from 3 independent experiments. p value (****<0.0001) was calculated using a Mann-Whitney test. n = 50 and 100 cells for control and 1.5% PEG groups, respectively. (E) Above: Schematic demonstrating protocol used for expansion and differentiation of human fetal lung distal lung tip progenitor cells. Below: Representative images of expanded cells, cells treated with AT2 differentiation media (AT2DM), and AT2DM + 1.5% PEG. (F) Quantification of % of Sftpc+ cells in AT2DM(Control) and AT2DM + 1.5% PEG conditions. P-value (**** < 0.0001) was calculated using a Mann-Whitney test. Supplemental Figure 2: (A) Representative images of pH Rhodo Dextran dye uptake assay of E16.5 DPs and nAT2 cells. (B) Quantification of fluid phase uptake by DP and nAT2. Statistical significance was determined using Student’s t-test yielding p-values of < 0.0001. (C) Transcriptional footprinting analysis of FGF signaling by using scRNA seq data of alveolar epithelium. (D) Schematic diagram of Fgfr2 downstream signaling target and their inhibition with small molecules [file NIHPP2026.03.04.708749V1-supplement-1.pdf]
